# *N*-(3,4-Dimethyl­phen­yl)benzamide

**DOI:** 10.1107/S1600536807066937

**Published:** 2007-12-21

**Authors:** B. Thimme Gowda, Miroslav Tokarčík, Jozef Kožíšek, B. P. Sowmya, Hartmut Fuess

**Affiliations:** a Department of Chemistry, Mangalore University, Mangalagangotri 574 199, Mangalore, India; b Faculty of Chemical and Food Technology, Slovak Technical University, Radlinského 9, SK-812 37 Bratislava, Slovak Republic; c Institute of Materials Science, Darmstadt University of Technology, Petersenstrasse 23, D-64287 Darmstadt, Germany

## Abstract

The conformation of the NH bond in the structure of the title compound (N34DMPBA), C_15_H_15_NO, is *anti* to the *meta*-methyl substituent in the aniline ring, similar to that observed with respect to the *meta*-chloro substituent in *N*-(3,4-dichloro­phen­yl)benzamide (N34DCPBA), but in contrast to the *syn* conformation observed with respect to the *meta*-methyl substituent in *N*-(3,4-dimethyl­phen­yl)acetamide. The bond parameters in N34DMPBA are similar to those in N34DCPBA and other benzanilides. The mol­ecules in N34DMPBA are packed into a column-like structure in the direction of the *a* axis through N—H⋯O hydrogen bonds.

## Related literature

For related literature, see: Gowda, Foro & Fuess (2007 ▶); Gowda *et al.* (2003 ▶); Gowda, Sowmya *et al.* (2007 ▶).
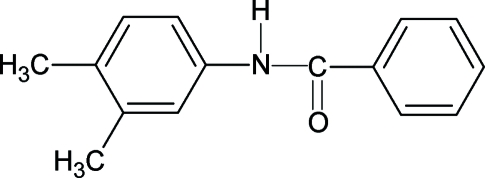


## Experimental

### 

#### Crystal data

C_15_H_15_NO
*M* *_r_* = 225.28
Orthorhombic, 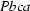

*a* = 9.1082 (2) Å
*b* = 9.8123 (2) Å
*c* = 28.5126 (8) Å
*V* = 2548.24 (10) Å^3^
*Z* = 8
Mo *K*α radiation
μ = 0.07 mm^−1^
*T* = 295 (2) K
0.33 × 0.11 × 0.08 mm

#### Data collection

Oxford Diffraction Xcalibur System diffractometer
Absorption correction: none
21605 measured reflections
2527 independent reflections
1448 reflections with *I* > 2σ(*I*)
*R*_int_ = 0.035

#### Refinement

*R*[*F* ^2^ > 2σ(*F* ^2^)] = 0.058
*wR*(*F* ^2^) = 0.194
*S* = 0.97
2527 reflections
159 parameters
3 restraints
H atoms treated by a mixture of independent and constrained refinement
Δρ_max_ = 0.37 e Å^−3^
Δρ_min_ = −0.19 e Å^−3^

### 

Data collection: *CrysAlis CCD* (Oxford Diffraction, 2007 ▶); cell refinement: *CrysAlis RED* (Oxford Diffraction, 2007 ▶); data reduction: *CrysAlis RED*; program(s) used to solve structure: *SHELXS97* (Sheldrick, 1997 ▶); program(s) used to refine structure: *SHELXL97* (Sheldrick, 1997 ▶); molecular graphics: *ORTEP-3* (Farrugia, 1997 ▶) and *DIAMOND* (Brandenburg, 2002 ▶); software used to prepare material for publication: *SHELXL97*, *PLATON* (Spek, 2003 ▶) and *WinGX* (Farrugia, 1999 ▶).

## Supplementary Material

Crystal structure: contains datablocks I, global. DOI: 10.1107/S1600536807066937/dn2302sup1.cif

Structure factors: contains datablocks I. DOI: 10.1107/S1600536807066937/dn2302Isup2.hkl

Additional supplementary materials:  crystallographic information; 3D view; checkCIF report

## Figures and Tables

**Table 1:** Hydrogen-bond geometry (Å, °)

*D*—H⋯*A*	*D*—H	H⋯*A*	*D*⋯*A*	*D*—H⋯*A*
N1—H1N⋯O1^i^	0.84 (2)	2.12 (2)	2.948 (2)	165 (2)

Symmetry code: (i) 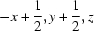.

**Fig. 1.**
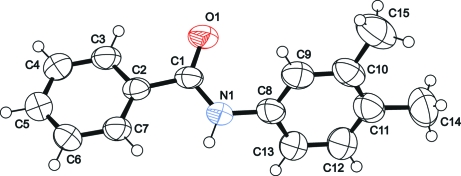
Molecular structure of the title compound showing the atom labelling scheme. Displacement ellipsoids are drawn at the 50% probability level. H atoms are represented as small spheres of arbitrary radii.

**Fig. 2.**
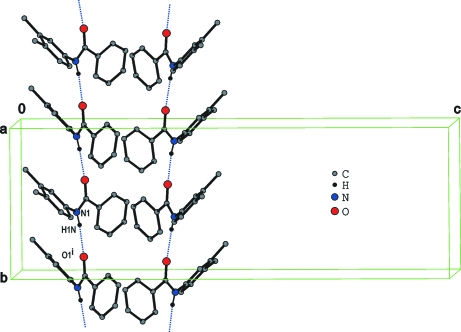
Part of crystal structure of the title compound showing the hydrogen bonds N1–H1N···O1(i). Symmetry code (i): -x + 1/2, y + 1/2, z.

## supplementary crystallographic information

### Comment

In the present work, the structure of *N*-(3,4-dimethylphenyl)-benzamide (N34DMPBA) has been determined to explore the effect of substituents on the structure of *N*-aromatic amides (Gowda *et al.*, 2003; Gowda, Sowmya *et al.*, 2007; Gowda, Foro & Fuess, 2007). The conformation of the N—H bond in N34DMPBA (FIg. 1) is anti to the *meta* methyl substituent in the aniline phenyl ring, similar to that observed with respect to the *meta* chloro substituent in *N*-(3,4-dichlorophenyl)-benzamide (N34DCPBA) (Gowda, Sowmya *et al.*, 2007), but in contrast to the *syn* conformation observed with respect to the *meta* methyl substituent in the *N*-(3,4-dimethylphenyl)- acetamide (Gowda, Foro & Fuess, 2007). The bond parameters in N34DMPBA are similar to those in N34DCPBA and other benzanilides (Gowda *et al.*, 2003). The molecules in N34DMPBA are packed into Column like s tructure in the direction of *a* axis through N—H···O hydrogen bonds (Table 1 & Fig. 2).

### Experimental

The title compound was prepared according to the literature method (Gowda *et al.*, 2003). The purity of the compound was checked by determining its melting point. It was characterized by recording its infrared and NMR spectra. Single crystals of the title compound were obtained from an ethanolic solution and used for X-ray diffraction studies at room temperature.

### Refinement

H atoms bonded to C atoms were placed in geometrically calculated positions and subsequently treated as riding with C–H distance 0.93Å for ring, 0.96Å for methyl. H(N) atom was visible in difference map. In the refinement the N–H distance was restrained to 0.86 (5) Å. The *U*_iso_(H) values were set at 1.2 *U*_eq_(C,*N*) of the parent atom (1.5 for methyl).

### Crystal data

**Table:**

C_15_H_15_NO	*F*_000_ = 960
*M**_r_* = 225.28	*D*_x_ = 1.174 Mg m^−^^3^
Orthorhombic, *P**b**c**a*	Mo *K*α radiation λ = 0.71073 Å
Hall symbol: -P 2ac 2ab	Cell parameters from 6829 reflections
*a* = 9.1082 (2) Å	θ = 3.0–29.5º
*b* = 9.8123 (2) Å	µ = 0.07 mm^−^^1^
*c* = 28.5126 (8) Å	*T* = 295 (2) K
*V* = 2548.24 (10) Å^3^	Prism, colourless
*Z* = 8	0.33 × 0.11 × 0.08 mm

### Data collection

**Table:**

Oxford Diffraction Xcalibur System diffractometer	2527 independent reflections
Radiation source: Enhance (Mo) X-ray Source	1448 reflections with *I* > 2σ(*I*)
Monochromator: graphite	*R*_int_ = 0.035
Detector resolution: 10.4340 pixels mm^-1^	θ_max_ = 26.2º
*T* = 295(2) K	θ_min_ = 5.1º
ω scans with κ offsets	*h* = −11→9
Absorption correction: none	*k* = −12→12
21605 measured reflections	*l* = −32→35

### Refinement

**Table:**

Refinement on *F*^2^	Secondary atom site location: difference Fourier map
Least-squares matrix: full	Hydrogen site location: inferred from neighbouring sites
*R*[*F*^2^ > 2σ(*F*^2^)] = 0.058	H atoms treated by a mixture of independent and constrained refinement
*wR*(*F*^2^) = 0.194	*w* = 1/[σ^2^(*F*_o_^2^) + (0.1315*P*)^2^] where *P* = (*F*_o_^2^ + 2*F*_c_^2^)/3
*S* = 0.97	(Δ/σ)_max_ < 0.001
2527 reflections	Δρ_max_ = 0.37 e Å^−^^3^
159 parameters	Δρ_min_ = −0.18 e Å^−^^3^
3 restraints	Extinction correction: none
Primary atom site location: structure-invariant direct methods	

### Special details

**Table:**

Geometry. All e.s.d.'s (except the e.s.d. in the dihedral angle between two l.s. planes) are estimated using the full covariance matrix. The cell e.s.d.'s are taken into account individually in the estimation of e.s.d.'s in distances, angles and torsion angles; correlations between e.s.d.'s in cell parameters are only used when they are defined by crystal symmetry. An approximate (isotropic) treatment of cell e.s.d.'s is used for estimating e.s.d.'s involving l.s. planes.
Refinement. Refinement of *F*^2^ against ALL reflections. The weighted *R*-factor *wR* and goodness of fit *S* are based on *F*^2^, conventional *R*-factors *R* are based on *F*, with *F* set to zero for negative *F*^2^. The threshold expression of *F*^2^ > σ(*F*^2^) is used only for calculating *R*-factors(gt) *etc*. and is not relevant to the choice of reflections for refinement. *R*-factors based on *F*^2^ are statistically about twice as large as those based on *F*, and *R*- factors based on ALL data will be even larger.

### Fractional atomic coordinates and isotropic or equivalent isotropic displacement parameters (Å^2^)

**Table:**

	*x*	*y*	*z*	*U*_iso_*/*U*_eq_	
C1	0.1683 (2)	0.51852 (17)	0.15178 (7)	0.0543 (5)	
C2	0.04493 (19)	0.58368 (18)	0.17772 (7)	0.0526 (5)	
C3	−0.0315 (2)	0.5050 (2)	0.21000 (8)	0.0651 (6)	
H3	−0.0063	0.4139	0.2141	0.078*	
C4	−0.1439 (3)	0.5599 (2)	0.23587 (9)	0.0762 (7)	
H4	−0.1931	0.5068	0.2578	0.091*	
C5	−0.1835 (3)	0.6937 (2)	0.22926 (9)	0.0766 (7)	
H5	−0.2602	0.7307	0.2466	0.092*	
C6	−0.1109 (2)	0.7724 (2)	0.19736 (9)	0.0724 (7)	
H6	−0.1384	0.8627	0.1929	0.087*	
C7	0.0038 (2)	0.71760 (19)	0.17159 (8)	0.0608 (6)	
H7	0.0534	0.7717	0.1500	0.073*	
C8	0.4041 (2)	0.55741 (19)	0.11157 (8)	0.0631 (6)	
C9	0.4082 (3)	0.4574 (2)	0.07867 (8)	0.0728 (7)	
H9	0.3209	0.4171	0.0690	0.087*	
C10	0.5432 (3)	0.4126 (2)	0.05859 (8)	0.0762 (7)	
C11	0.6702 (3)	0.4749 (2)	0.07412 (9)	0.0804 (7)	
C12	0.6653 (3)	0.5762 (3)	0.10680 (10)	0.0874 (8)	
H12	0.7520	0.6175	0.1165	0.105*	
C13	0.5336 (2)	0.6185 (3)	0.12574 (10)	0.0751 (7)	
H13	0.5319	0.6878	0.1480	0.090*	
C14	0.8200 (4)	0.4325 (3)	0.05424 (14)	0.1195 (12)	
H14A	0.8943	0.4939	0.0655	0.179*	
H14B	0.8169	0.4358	0.0206	0.179*	
H14C	0.8426	0.3415	0.0642	0.179*	
C15	0.5398 (4)	0.3011 (3)	0.02331 (12)	0.1179 (11)	
H15A	0.6176	0.3142	0.0010	0.177*	
H15B	0.4471	0.3020	0.0073	0.177*	
H15C	0.5525	0.2150	0.0388	0.177*	
N1	0.26924 (19)	0.60131 (17)	0.13237 (7)	0.0614 (5)	
H1N	0.268 (2)	0.685 (2)	0.1389 (8)	0.074*	
O1	0.17788 (16)	0.39356 (13)	0.14930 (6)	0.0777 (5)	

### Atomic displacement parameters (Å^2^)

**Table:**

	*U*^11^	*U*^22^	*U*^33^	*U*^12^	*U*^13^	*U*^23^
C1	0.0539 (11)	0.0379 (10)	0.0710 (13)	−0.0013 (8)	−0.0095 (9)	0.0014 (8)
C2	0.0473 (11)	0.0428 (11)	0.0677 (13)	−0.0043 (7)	−0.0114 (9)	0.0008 (8)
C3	0.0660 (14)	0.0438 (11)	0.0854 (15)	−0.0053 (9)	−0.0009 (11)	0.0047 (10)
C4	0.0789 (16)	0.0630 (14)	0.0869 (16)	−0.0100 (11)	0.0189 (13)	0.0031 (11)
C5	0.0701 (15)	0.0670 (15)	0.0927 (17)	0.0000 (11)	0.0149 (12)	−0.0082 (12)
C6	0.0709 (14)	0.0501 (11)	0.0963 (16)	0.0082 (10)	0.0072 (13)	−0.0006 (11)
C7	0.0604 (12)	0.0439 (11)	0.0781 (14)	−0.0018 (9)	0.0001 (10)	0.0058 (10)
C8	0.0752 (15)	0.0450 (11)	0.0692 (13)	0.0032 (10)	0.0047 (11)	0.0064 (10)
C9	0.0859 (17)	0.0577 (13)	0.0748 (14)	−0.0017 (11)	−0.0030 (12)	0.0058 (11)
C10	0.107 (2)	0.0519 (13)	0.0697 (15)	0.0122 (12)	0.0034 (13)	0.0056 (11)
C11	0.0936 (19)	0.0621 (14)	0.0855 (16)	0.0091 (12)	0.0067 (14)	0.0044 (12)
C12	0.0704 (16)	0.0881 (17)	0.1036 (19)	0.0007 (13)	0.0041 (14)	−0.0054 (15)
C13	0.0639 (14)	0.0710 (15)	0.0903 (17)	−0.0011 (11)	0.0054 (12)	−0.0065 (12)
C14	0.106 (2)	0.108 (2)	0.145 (3)	0.0300 (18)	0.039 (2)	0.0006 (19)
C15	0.174 (3)	0.085 (2)	0.095 (2)	0.0096 (18)	0.011 (2)	−0.0201 (16)
N1	0.0628 (11)	0.0394 (9)	0.0821 (12)	0.0001 (8)	0.0098 (9)	−0.0019 (8)
O1	0.0706 (10)	0.0401 (9)	0.1224 (14)	0.0007 (6)	0.0090 (9)	0.0026 (8)

### Geometric parameters (Å, °)

**Table:**

C1—O1	1.231 (2)	C9—C10	1.425 (4)
C1—N1	1.346 (2)	C9—H9	0.9300
C1—C2	1.490 (3)	C10—C11	1.382 (4)
C2—C7	1.377 (3)	C10—C15	1.487 (4)
C2—C3	1.389 (3)	C11—C12	1.363 (4)
C3—C4	1.372 (3)	C11—C14	1.535 (4)
C3—H3	0.9300	C12—C13	1.379 (4)
C4—C5	1.374 (3)	C12—H12	0.9300
C4—H4	0.9300	C13—H13	0.9300
C5—C6	1.364 (3)	C14—H14A	0.9600
C5—H5	0.9300	C14—H14B	0.9600
C6—C7	1.386 (3)	C14—H14C	0.9600
C6—H6	0.9300	C15—H15A	0.9600
C7—H7	0.9300	C15—H15B	0.9600
C8—C9	1.358 (3)	C15—H15C	0.9600
C8—C13	1.384 (3)	N1—H1N	0.84 (2)
C8—N1	1.430 (3)		
			
O1—C1—N1	121.96 (18)	C11—C10—C9	117.2 (2)
O1—C1—C2	120.61 (17)	C11—C10—C15	124.1 (3)
N1—C1—C2	117.41 (15)	C9—C10—C15	118.7 (3)
C7—C2—C3	118.57 (18)	C12—C11—C10	120.9 (2)
C7—C2—C1	123.48 (17)	C12—C11—C14	118.6 (3)
C3—C2—C1	117.95 (16)	C10—C11—C14	120.4 (3)
C4—C3—C2	120.78 (19)	C11—C12—C13	121.0 (2)
C4—C3—H3	119.6	C11—C12—H12	119.5
C2—C3—H3	119.6	C13—C12—H12	119.5
C3—C4—C5	119.8 (2)	C12—C13—C8	119.8 (2)
C3—C4—H4	120.1	C12—C13—H13	120.1
C5—C4—H4	120.1	C8—C13—H13	120.1
C6—C5—C4	120.3 (2)	C11—C14—H14A	109.5
C6—C5—H5	119.8	C11—C14—H14B	109.5
C4—C5—H5	119.8	H14A—C14—H14B	109.5
C5—C6—C7	120.0 (2)	C11—C14—H14C	109.5
C5—C6—H6	120.0	H14A—C14—H14C	109.5
C7—C6—H6	120.0	H14B—C14—H14C	109.5
C2—C7—C6	120.50 (19)	C10—C15—H15A	109.5
C2—C7—H7	119.7	C10—C15—H15B	109.5
C6—C7—H7	119.7	H15A—C15—H15B	109.5
C9—C8—C13	119.4 (2)	C10—C15—H15C	109.5
C9—C8—N1	121.9 (2)	H15A—C15—H15C	109.5
C13—C8—N1	118.7 (2)	H15B—C15—H15C	109.5
C8—C9—C10	121.6 (2)	C1—N1—C8	125.11 (16)
C8—C9—H9	119.2	C1—N1—H1N	119.4 (16)
C10—C9—H9	119.2	C8—N1—H1N	113.3 (16)
			
O1—C1—C2—C7	−161.5 (2)	C8—C9—C10—C15	179.0 (2)
N1—C1—C2—C7	19.9 (3)	C9—C10—C11—C12	−0.9 (4)
O1—C1—C2—C3	19.0 (3)	C15—C10—C11—C12	−179.6 (2)
N1—C1—C2—C3	−159.59 (19)	C9—C10—C11—C14	180.0 (2)
C7—C2—C3—C4	−1.2 (3)	C15—C10—C11—C14	1.3 (4)
C1—C2—C3—C4	178.3 (2)	C10—C11—C12—C13	0.8 (4)
C2—C3—C4—C5	1.3 (4)	C14—C11—C12—C13	179.9 (3)
C3—C4—C5—C6	−0.5 (4)	C11—C12—C13—C8	0.1 (4)
C4—C5—C6—C7	−0.3 (4)	C9—C8—C13—C12	−0.7 (3)
C3—C2—C7—C6	0.4 (3)	N1—C8—C13—C12	178.9 (2)
C1—C2—C7—C6	−179.1 (2)	O1—C1—N1—C8	−7.0 (3)
C5—C6—C7—C2	0.3 (3)	C2—C1—N1—C8	171.52 (18)
C13—C8—C9—C10	0.6 (3)	C9—C8—N1—C1	51.0 (3)
N1—C8—C9—C10	−179.02 (19)	C13—C8—N1—C1	−128.6 (2)
C8—C9—C10—C11	0.2 (3)		

### Hydrogen-bond geometry (Å, °)

**Table:**

*D*—H···*A*	*D*—H	H···*A*	*D*···*A*	*D*—H···*A*
N1—H1N···O1^i^	0.84 (2)	2.12 (2)	2.948 (2)	165 (2)

Symmetry codes: (i) −*x*+1/2, *y*+1/2, *z*.
